# Liver resection for hepatocellular carcinoma in patients with hematological malignancies

**DOI:** 10.1186/s12957-017-1260-y

**Published:** 2017-11-02

**Authors:** Hui-Chen Lin, Yang-Shen Yang, Chieh-Lin Jerry Teng, Ching-Hui Shen, Yee-Gee Jan, Shao-Bin Cheng, Cheng-Chung Wu, Yi-Ling Lin, Chu-Chun Huang, Fang-Ku P’eng

**Affiliations:** 1 Department of Surgery, Taichung Veterans General Hospital, Section, 4, No. 1650, Taiwan Boulevard, Taichung, Taiwan; 2 Department of Hematology, Taichung Veterans General Hospital, Taichung, Taiwan; 3 Department of Anesthesiology, Taichung Veterans General Hospital, Taichung, Taiwan; 4 Department of Pathology, Taichung Veterans General Hospital, Taichung, Taiwan; 5 Department of Surgery, School of Medicine, National Yang-Ming University, Taipei, Taiwan; 6 Department of Surgery, Taipei Medical University, Taipei, Taiwan; 7 Department of Surgery, Chung-Shan Medical University, Taichung, Taiwan

**Keywords:** Hepatocellular carcinoma, Liver resection, Hematological malignancy

## Abstract

**Background:**

Improvements in antimetabolite drugs have prolonged the survival of patient with hematological malignancies. However, these drugs may have hepatotoxic side effects and may induce acute liver failure, chronic liver fibrosis, cirrhosis, or even hepatocellular carcinoma (HCC). Although liver resection remains a curative option for HCC, its role in HCC with hematological malignancies has never been fully explored.

**Methods:**

A retrospective review of 1725 patients who underwent curative liver resection for newly diagnosed HCC between 1994 and 2016 was conducted. Among these patients, 16 had a history of hematological malignancies (HM group). Their hematological malignancies were well-controlled at the time of liver resection. The clinicopathological characteristics of the HM group, along with their short- and long-term outcomes after liver resection, were compared with those of the other 1709 patients without hematological malignancy (non-HM group).

**Results:**

All HM group patients were seropositive for hepatitis marker surface for hepatitis B and C. No significant differences were observed in any background characteristics between the two groups. The postoperative complication rate and 90-day mortality in the HM and non-HM groups were 25 and 20.4%, *P* = 0.754, and 0 and 0.6%, *P* = 1.000, respectively. The 5-year disease-free and overall survival rates for the HM and non-HM groups were 42.3 and 35.1%, *P* = 0.552, and 69.5 and 56.9%, *P* = 0.192, respectively.

**Conclusions:**

Hepatitis markers should be examined during chemotherapy for hematological malignancies. Regular liver imaging studies are recommended for seropositive cases. When HCC occurs secondary to a well-controlled hematological malignancy, liver resection is suggested in selected patients.

## Background

Outcomes of hematological malignancies have improved substantially and survival times have markedly increased due to the progression of antimetabolite therapies [1]. However, the hepatotoxic side effects of antimetabolite drugs are an important consideration [2–5]. Acute hepatocyte necrosis and fulminant liver failure may occur after administration of these agents, especially in patients with chronic hepatitis B or C infection [2, 3]. Moreover, antimetabolite drugs may cause methylation of mitochondrial DNA in hepatocytes, resulting in phenotypic changes, and may induce malignant change in hepatocytes leading to subsequent primary hepatocellular carcinoma (HCC) formation [4, 5]. Therefore, liver fibrosis, cirrhosis, and HCC have been reported after long-term use of these drugs [4]. Moreover, as Taiwan is an endemic area of hepatitis B, HCC has consistently ranked as the first or second leading cause of cancer death in the past 20 years. Most HCC patients in Taiwan are seropositive for hepatitis B surface antigen (HBsAg) and/or anti-hepatitis C antibody (anti-HCV) [6–16]. When patients with hepatitis also have hematological malignancies, the hepatocytes that have been injured by hepatitis B or C may be more susceptible to antimetabolite-induced insults and accelerated HCC formation [4–8]. We have observed the development of HCC in patients with hematological malignancies.

At present, radiofrequency ablation (RFA), liver resection, and liver transplantation are well-recognized as curative options for HCC [6, 7]. However, these strategies have their advantages and limitations. Appropriate case selection with multimodality therapies may provide long-term survival and optimal quality of life [6–8]. Among these strategies, liver resection remains the most widely adopted classical curative modality for HCC [9–18]. However, liver resection is a highly complex procedure with a high complication rate despite its low mortality in high-volume centers [9].

To the best of our knowledge, the role of liver resection for HCC in patients with hematological malignancies has never been investigated. In this study, a retrospective analysis of prospectively collected data from HCC patients who underwent liver resection was conducted. The aim of the study was to elucidate the benefits of liver resection for HCC in this patient cohort.

## Methods

This study was approved by the institutional review board (IRB). A retrospective review of prospectively collected data from 1725 consecutive patients who underwent curative liver resection for newly diagnosed HCC between 1994 and 2016 was conducted. Curative resection was defined as the removal of tumors that were preoperatively detected by imaging studies or the removal of tumors that were detected intraoperatively by palpation or ultrasonography [10–18].

Among the HCC patients, 16 (0.9%) had a history of varied hematological malignancies. All patients received generally recommended regimens of systemic chemotherapies [1]. The HCCs in 3 patients were detected during the course of chemotherapy, while the HCCs in the other 13 patients were detected at an average of 79 (range, 9–174) months after hematological treatment. The hematological malignancies included large B-cell lymphoma in eight patients (nodal type 5, extranodal type 3 (stomach, 1, orbit floor 1, colon 1), multiple myeloma in three, chronic myelocytic leukemia in two patients, chronic lymphocytic leukemia in two patients, and acute myelocytic leukemia in one patient. For the three HCCs (one lymphoma, multiple myeloma, and chronic lymphocytic leukemia) that were found during the course of chemotherapy, the chemotherapy (synchronous HCC) was temporarily discontinued for 4 weeks and was then re-started 2 weeks after total recovery from the hepatectomy or after complete postoperative healing had occurred. The policies and protocols of the systemic anticancer therapies for these hematological malignancies are listed in Table 1. Two patients had ≥ 3 malignancies (lymphoma, papillary thyroid carcinoma, stomach cancer and HCC in 1 patient; multiple myeloma, basal cell carcinoma of the chest wall, and HCC in 1 patient).

**Table 1:** Anticancer drugs for hematological malignancies

Hematologic malignancy	Anticancer drugs
Non-Hodgkin’s lymphoma	
Slowly indolent lymphoma	Modest chemotherapy
Aggressive lymphoma	Intensive chemotherapy
Diffuse large B cell lymphoma	Standard first-line chemotherapy
	Rituximab plus CHOP (cyclophosphamide, vincristine, doxorubicin, prednisolone)
Multiple myeloma	1st-line thalidomide; ASCT (autologous stem cell transplant)
	2nd-line bortezomib (Velcade), thalidomide, dexamethasone
	Lenalidomide
Chronic lymphocytic leukemia	CVP (cyclophosphamide, vincristine, prednisolone)
	Bendamustine, rituximab, fludarabine
Acute myeloid leukemia	Cytosine arabinoside
	Idarubicin

### Patient selection criteria for HCC resection

#### Assessments of HCC patients who underwent liver resection

The hematological malignancies of all patients were considered to be well-controlled by hematologists (YSY and JLT). The extrahepatic malignancies in two patients who had ≥ 3 primary malignancies were also considered to be controlled at the time of HCC diagnosis. The results of conventional liver function tests, seropositivity for HBsAg and anti-HCV, and the serum α-fetoprotein (AFP) level were determined [9–15]. The indocyanine-green 15-min retention rate (ICG R15) was determined for each patient. Child-Pugh grading for cirrhotic liver function [16] was conducted for all patients. If the patient’s general condition fulfilled the criteria for American Society of Anesthesiology (ASA) class 1 or 2, liver resection was indicated [9]. Computed tomography (CT) or magnetic resonance imaging (MRI) of the liver was performed in all patients. The extent of liver resection was based on the ICG R15, and tumor extension was estimated using a modified version of the Makuuchi criteria for cirrhotic liver resection [9, 11–15]. Concomitant splenectomy was performed if the patient had hypersplenic thrombocytopenia (platelet count < 8 × 10^4^/mm^3^) [13]. Preoperative gastroduodenal endoscopy was performed in all patients. If severe gastro-esophageal varices were found, they were treated by endoscopic sclerotherapy or variceal ligation [14]. Perioperative heparin-free hemodialysis was performed for patients with end-stage renal disease (from 1 day before surgery to postoperative day 7) [15].

During surgery, intraoperative ultrasonography was routinely performed to define the location of major intrahepatic vessels and tumors that were not visible or impalpable. The liver parenchyma was transected using the Kelly clamp crush technique with intermittent hepatic inflow blood occlusion under low central venous pressure (approximately 5 cm H_2_O), which was maintained by a senior anesthesiologists. A limited blood transfusion policy was applied to all patients.

The resected specimens were sent to a pathologist (YGJ) to determine the pathologic characteristics of HCC. UICC-TNM tumor staging (8th edition) [17] was applied to each patient for tumor staging after pathological examination. Cirrhosis severity was assessed using the Ishak scoring system [18].

Operative mortality was defined as a death within 90 days postoperatively. The Clavien-Dindo complication severity [19] was also evaluated in patients with complications. After undergoing hepatectomy, patients were followed up at both surgery and hematology out-patient clinics. If HCC recurred, patients were treated by re-resection, radiofrequency ablation (after 2000, for tumor with ≤ 3 nodules, each ≤ 3 cm), transarterial chemoembolization (TACE) [19], Sorafenib (after 2009 in our hospital) or conservative treatment as appropriate.

### Statistical analysis

The clinicopathological characteristics of the 16 patients with a history of hematologic malignancies (HM group) were compared with those of the other 1709 patients without hematologic malignancies (non-HM group). The early postoperative results and the long-term disease-free (DFS) and overall survival (OS) rates were also compared between the two groups.

Continuous variables are expressed as medians (ranges) and were compared using the Mann-Whitney *U* test. Frequencies were compared using Fisher’s exact test or Pearson’s *χ* ^2^ test as appropriate. All patients were followed up until June 2017. The Kaplan-Meier life table method was used to calculate the DFS and OS rates. The log-rank test was used to compare the difference in survival between the two groups.

## Results

The clinical characteristics of all patients who underwent liver resection are shown in Table 2 [20]. No significant differences were observed in any of the variables between the two groups. Notably, all patients in the HM group were seropositive for HBsAg and/or anti-HCV, although the data were not significantly different from those of patients in the non-HM group.

**Table 2:** Background characteristics of HCC patients who underwent hepatectomy

Characteristics	HM group (*n* = 16)	Non-HM group (*n* = 1709)	*P* value
Sex (male:female)	11 (68.8%):5 (31.2%)	1009 (59.0%):700 (41.0%)	0.595
Age	64 (32–80)	61.5 (18–95)	0.877
Hepatitis status			0.815
B+ C+	2 (12.5%)	82 (4.0%)	
B+ C−	8 (50.0%)	784 (46.1%)	
B− C+	6 (37.5%)	579 (34.1%)	
B− C−	0 (0%)	264 (14.9%)	
AFP (ng/ml)	18.4 (5.19–189)	27.2 (19–10,906)	0.097
Cirrhosis	14 (87.5%)	1271 (75.2%)	0.384
ICG R15 (%)	16.4 (8.9–271)	13.6 (5.1–50.1)	0.472
Splenectomy	2 (12.5%)	130 (7.7%)	0.231
Association with GEV	1 (6.7%)	211 (12.4%)	0.375
Comorbidities (other than HCC and hematologic malignancies)			
Heart disease	1	114	1.0
Respiratory disease	0	54	1.0
Uremia	0	54	1.0
Old stroke	1	47	0.365
AIDS	0	5	1.0
Malnutrition	0	4	1.0
Heavy-metal intoxication	0	2	1.0
Alcohol overintake^a^	1	102	1.0
Lupus erythematosus	1	11	0.106

*B+ C+* seropositive for HBsAg and AntiHCV; *B+ C−* seropositive for HBsAg, seronegative for AntiHCV; *B− C+* seronegative for HBsAg, seropositive for AntiHCV; *B− C−* seronegative for HBsAg and AntiHCV; *ICG R15* ICG 15-min retention rate; *GEV* gastroesophageal varices

^a^Defined as daily alcohol intake ≥ 20 g [20]

Early post-operative results are shown in Table 3. No significant differences were found for any complications or severities (Clavien-Dindo classification^17^) between the two groups. The 90-day incidence of mortality after surgery in the HM and non-HM groups were 0 and 11 (0.6%) (*P* = 1.000), respectively.

**Table 3:** Early postoperative result after hepatectomy

	HM group (*n* = 16)	Non-HM group (*n* = 1709)	*P* value
Total Pringle clamping (min)	45.0 (23.6–84.6)	59.6 (0–168.8)	0.112
Operation time (h)	4.58 (3.85–5.42)	4.17 (3.5–11.14)	0.328
Op blood loss (ml)	625 (340–7500)	500 (50–8500)	0.545
No blood transfusion	13 (81.3%)	1467 (86.0%)	0.482
Postop stay (day)	9 (7–29)	8 (7–48)	0.774
Complication	4 (25.0%)	341 (20.4%)	0.754
Bile leakage	2	160	
Abdominal abscess	1	104	
Ascites	1	94	
Wound infection	0	57	
Postop bleeding	0	31	
Prolonged jaundice	0	40	
MI	0	11	
Stroke	0	4	
Clavien-Dindo grading			0.788
Grade 3	1	82	
Grade 4	1	29	
Grade 5	0	11	
90-day mortality	0 (0.0%)	11 (0.6%)	1.000

*Op* operative, *Postop* postoperative

The pathological characteristics of the resected specimens are listed in Table 4. No significant differences were found between the two groups.

**Table 4:** Pathological characteristics of the resected HCC

	HM group (*n* = 16)	Non-HM group (*n* = 1709)	*P* value
Extent of liver resection			0.925
≤ 1 segment*	10 (62.5%)	1031 (61.5%)	
1.1–2.9 segments*	4 (25.0%)	403 (24.0%)	
≥ 3 segments*	2 (12.5%)	242 (14.1%)	
Resected liver weights (g)	135 (48.8–1200)	105 (50–1600)	0.818
Tumor size (cm)	4.6 (2.6–8.0)	5.2 (3–17)	0.921
Tumor number ≥ 2	3 (18.8%)	317 (18.9%)	0.999
Presence of vascular invasion	8 (50%)	994 (58.2%)	0.918
Presence of *satellite nodules*	6 (37.5%)	772 (45.2%)	0.890
Presence of capsule	9 (56.3%)	899 (52.8%)	0.982
TNM stage^#^			0.815
I	8 (50%)	687 (39.0%)	
II	4 (25%)	515 (30.6%)	
III	4 (25%)	493 (29.2%)	
IV	0 (00%)	20 (1.2%)	
Tumor differentiation			0.903
WD	2	197	
MD + PD	14	502	
Resection margin (mm)	5 (1.5–14.0)	3 (0–19.0)	

*Couinaud’s Segment ^#^7th edition (2009)

**Fig. 1:**
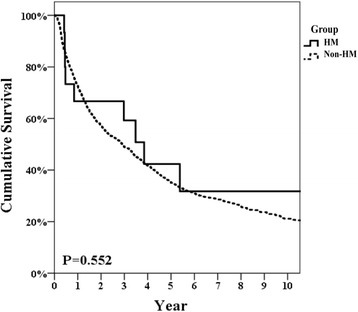
Disease-free survival rate of HCC patients after resection

**Fig. 2:**
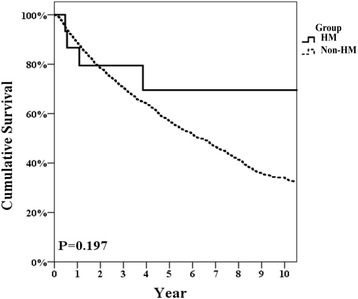
Overall survival rate of HCC patients after resection

Repeat hepatectomy was performed on two patients in the HM group and on 298 patients in the non-HM group. As shown in Fig. 1, the 2-, 5-, and 10-year-DFS rates for HCC in the HM group were 66.7, 42.3, and 31.7%, while the 2-, 5-, and 10-year -DFS rates for HCC in the non-HM group were 57.2, 35.1, and 21.1%, respectively (*P* = 0.552). Figure 2 shows that the 2-, 5-, and 10-year-OS rates after resection of HCC in the HM group were 79.4, 69.5, and 69.5%, while the 2-, 5-, and 10-year-OS rates for HCC in the non-HM group were 78.4, 56.9, and 34.1%, respectively (*P* = 0.197). No patients in the HM group had a recurrence of or died from a hematological malignancy. One 53-year-old HBsAg-positive woman underwent S8 liver resection of a 6-cm HCC (AJCC-TNM stage T2N0M0) on February 12, 2006. She underwent total thyroidectomy for papillary carcinoma of the thyroid in 1990. She also underwent a D2 distal gastrectomy for gastric carcinoma in 1993 (AJCC-TNM stage T4aN0M0). She had a malignant lymphoma (B-cell type) in a neck lymph node and underwent chemotherapy in 1995. An S6 recurrent HCC was resected on December 16, 2011. Neck lymphatic recurrence of thyroid cancer was diagnosed in July 2013, and a modified neck dissection with radio-iodine ablation was performed. She remains disease-free at the time of this report, 11 years after the first hepatectomy and 5.5 years after the second hepatectomy.

## Discussion

In this study, HCC patients with hematological malignancies (HM group) were categorized as having a multiple primary malignancy (MPM) disease. The term MPM was first proposed by Billroth in 1884 [21]. In 1932, Warren and Gates conducted a literature review that included autopsy cases and found evidence of more than 1000 cases of MPM [22]. With recent improvements in diagnostic tools and prolonged survival rates after malignancy treatment, the incidence of MPM has gradually increased. Berrington de Gonzalez, et al. [23] reported that 18% of cancer patients who had received radiotherapy in the USA had MPM. In the European Union, the incidence of MPM was reported to be 11% [24]. For MPM that included HCC, a study conducted in Korea in 2012 reported an incidence rate of 11.5% [25].

The cause of secondary liver malignancy formation in patients with hematologic malignancy is not well-recognized. In our series, not surprisingly, hepatitis B and C still played important roles in HCC formation in HM group patients, as all patients were seropositive for HBsAg and/or anti-HCV. A possible mechanism involves hepatocyte injury (either acute or chronic) by antimetabolite drugs, which can cause genetic changes and cancer cell formation. All hematologic malignancies developed in patients whose conditions were well-controlled. Taiwan is an endemic area for hepatitis B virus; a large proportion of the general population is seropositive for HBsAg. When affected individuals receive antimetabolite drugs, the hepatotoxic side effects of these drugs may cause liver cell damage or liver cell carcinogenesis by methylation of mitochondria [4–7]. In the past, physicians in our institution reported the use of large doses of antimetabolite drugs during pulse therapy for active collagen disease with massive liver necrosis requiring liver transplantation [2]. Thereafter, they established a guideline to perform routine evaluations of liver function and serum HBsAg and anti-HCV seropositivity for the early detection of liver injury after chemotherapy [3].

In this series, all HM group patients were seropositive for hepatitis markers. As with general HCC populations who have a high incidence of positive serum hepatitis markers, regular liver imaging studies should be mandatory in HBsAg- or anti-HCV-positive patients undergoing chemotherapy for hematologic malignancies to determine the presence of liver tumors. Moreover, HCC formation and hepatotoxic conditions such as acquired immunodeficiency syndrome (AIDS), malnutrition, alcohol overintake (defined as daily alcohol intake ≥ 20 g [20]), and heavy metal toxicity can be researched using present study model. Nevertheless, the incidence of these conditions in our HCC patients are very low (Table 2). Yet, the true mechanism of HCC formation in these circumstances remained unknown.

The outcome of MPM patients remains controversial. Long-term survival could be achieved in patients whose malignancies are well-controlled. The prognosis of unresectable HCC in patients with hematological malignancy is usually dismal [4]. The association of HCC and hematological malignancy is unique; the incidence was only 0.93% in our series over 22 years. A report from Beijing, China, in which a 26-year follow-up study was conducted, reported that the most common secondary cancers with HCC were lung cancer, colorectal cancer, and thyroid cancer. Only 1 of 40 HCC patients had a secondary site that indicated blood cancer. The long-term results of liver resection of HCC with extrahepatic secondary malignant tumors were similar to those of HCC patients without secondary cancers [26]. Most of these patients succumbed to HCC recurrence. Similar results were reported in a study conducted in southern Japan [27], which found that most cancer recurrences consisted of HCC after liver resection for HCC and extrahepatic secondary malignancies.

Our hematologists routinely monitored patients by performing liver imaging studies during or after treatment for hematologic malignancies. For controlled hematological malignancies, aggressive liver resection for HCC yielded similar early and long-term outcomes as general resectable HCC cases.

The resectability of HCC depends on the patient’s general condition and liver function and the tumor location. In the HM group, chemotherapy for hematological malignancy may induce decreased host immunity. Thus, the risk of liver resection in these patients may increase. However, the patient selection criteria for HCC in the non-HM group were similar to those applied in the HM group. No operative mortality occurred in the HM group, and the postoperative morbidity was also acceptable. The surgical risks were similar in the HM and non-HM group, particularly in patients whose HCCs were detected during chemotherapy. We believe that if hematological malignancies can be well-controlled, liver resection is still safe in selected patients. HCC resection after prior chemotherapy for hematological malignancy was not a particularly influential factor.

The long-term outcomes of our patients were also affected by the recurrence of HCC, but no recurrence of hematological malignancies was observed; no patient in our series had a recurrent or uncontrolled (progression) hematological malignancy. Therefore, post-operative follow-up of these patients should be especially mindful of the possibility of HCC recurrence, in addition to checking for any evidence of hematological malignancies. If there is suspicion of HCC recurrence, multidisciplinary therapies should be urgently applied. With respect to the OS in this study, we found that the DFS and OS of HCC patients were similar to those of the general HCC population.

## Conclusions

In conclusion, follow-up after treatment for hematological malignancy should include hepatitis marker, liver functions biomarker, and imaging studies to detect HCC occurrence especially in patients who are positive for HBsAg or anti-HCV. Once HCC has been detected, aggressive liver resection is recommended for selected patients.

## Abbreviations

AFP: α-Fetoprotein
Anti-HCV: Anti-hepatitis C antibody
ASA: American Society of Anesthesiology
CT: Computed tomography
DFS: Disease-free survival
HBsAg: Hepatitis B surface antigen
HCC: Hepatocellular carcinoma
HM: Hematologic malignancies
ICG R15: Indocyanine-green 15-min retention rate
IRB: Institutional review board
MRI: Magnetic resonance imaging
OS: Overall survival
RFA: Radiofrequency ablation
TACE: Transarterial chemoembolization
